# Role of HIV in the desire of procreation and motherhood in women living with HIV in Spain: a qualitative approach

**DOI:** 10.1186/s12905-017-0483-y

**Published:** 2018-01-24

**Authors:** Debora Alvarez-del Arco, Sabela Rodríguez, Mª. Jesús Pérez-Elías, Jose Ramón Blanco, Sandra Cuellar, Jorge del Romero, Ignacio Santos, Vicente Boix, Mar Masiá, Lydia Pascual, Victoria Hernando, CoRIS, Alicia Llacer, Santiago Moreno, Julia del Amo, David Dalmau, Maria Luisa Navarro, Maria Isabel González-Tomé, Federico García, José Luis Blanco, Rafael Rubio, Jose Antonio Iribarren, Francesc Vidal, Félix Gutiérrez, Juan Berenguer, Juan González, Paz Sobrino Vegas, Victoria Hernando Sebastián, Belén Alejos Ferreras, Débora Álvarez-del Arco, Yaiza Rivero, Inmaculada Jarrín, M. Ángeles Muñoz-Fernández, Isabel García-Merino, Coral Gómez Rico, Jorge Gallego de la Fuente, Almudena García Torre, Joaquín Portilla, Esperanza Merino, Sergio Reus, Vicente Boix, Livia Giner, Carmen Gadea, Irene Portilla, Maria Pampliega, Marcos Díez, Juan Carlos Rodríguez, Jose Sánchez-Payá, José Antonio Iribarren, Julio Arrizabalaga, María José Aramburu, Xabier Camino, Francisco Rodríguez-Arrondo, Miguel Ángel von Wichmann, Lidia Pascual Tomé, Miguel Ángel Goenaga, Mª. Jesús Bustinduy, Harkaitz Azkune Galparsoro, Maialen Ibarguren, Miriam Aguado, Mar Masiá, Cristina López, Sergio Padilla, Andrés Navarro, Fernando Montolio, Catalina Robledano, Joan Gregori Colomé, Araceli Adsuar, Rafael Pascual, Federico Carlos, Maravillas Martínez, Marta Montero, José López Aldeguer, Marino Blanes, José Lacruz, Miguel Salavert, Eva Calabuig, Sandra Cuéllar, Ignacio de los Santos, Jesús Sanz, Ana Salas, Cristina Sarriá, Ana Gómez, José Antonio Oteo, José Ramón Blanco, Valvanera Ibarra, Luis Metola, Mercedes Sanz, Laura Pérez-Martínez, José Luis Casado, Fernando Dronda, Ana Moreno, María Jesús Pérez Elías, Dolores López, Carolina Gutiérrez, Nadia Madrid, Angel Lamas, Paloma Martí, Alberto de Diaz, Sergio Serrano, Jorge Del Romero Guerrero, Carmen Rodríguez Martín, Teresa Puerta López, Juan Carlos Carrió Montiel, Mar Vera

**Affiliations:** 1 Red de Investigación en Sida, Centro Nacional de Epidemiología, Instituto de Salud Carlos III, Avenida Monforte de Lemos 5, Madrid, Spain; 2 CIBER de Epidemiología y Salud Pública (CIBERESP), Madrid, Spain; 3 Departament of Sociology IV, Universidad Complutense de Madrid, Somosaguas, Madrid, Spain; 4 Hospital Universitario Ramón y Cajal, Madrid, Spain; 5 Hospital Universitario San Pedro-CIBIR, Logroño, Spain; 6 Hospital Universitario La Fe, Valencia, Spain; 7 Centro Sanitario Sandoval, Madrid, Spain; 8 Hospital Universitario La Princesa, Madrid, Spain; 9 Hospital Universitario de Alicante, Alicante, Spain; 10 Hospital Universitario de Elche, Elche, Spain; 11 Hospital Universitario Donostia, Donostia, Spain

## Abstract

**Background:**

Improved antiretroviral treatments and decrease in vertical transmission of HIV have led to a higher number of women living with HIV to consider childbearing. However, stigma and social rejection result in specific challenges that HIV positive women with procreation intentions have to face with. Our objective was to in depth analyse elements shaping their desire for procreation and specifically investigate the impact of HIV.

**Methods:**

A qualitative study was conducted through open interviews with 20 women living with HIV between 18 and 45 years of age, from the Spanish AIDS Research Network Cohort (CoRIS). Interviews were audio-recorded and transcribed. A content analysis was performed.

**Results:**

HIV diagnosis is a turning point in women’s sexual and emotional life that is experienced traumatically. HIV diagnosis is usually associated with the fear of an immediate death and the idea of social isolation. At this moment, women temporarily reject future motherhood or having a sexual life. HIV status is only disclosed to the closed social circle and partner support is essential in HIV diagnosis assimilation process. Health professionals provide information on assisted reproductive technology and on how to minimize risk of partner HIV transmission. Most of barriers for procreation acknowledged by women are not related to HIV. However, women fear vertical transmission and experience other barriers derived from HIV infection. In this context, pregnancy makes women feel themselves as “normal women” despite HIV. Motherhood is considered an element of compensation that helps them to cope with HIV diagnosis. All these elements make health professionals key actors: they provide information and support after HIV diagnosis.

**Conclusions:**

Barriers and drivers for procreation are similar among HIV positive women and general population. However, stigma and discrimination linked with HIV weigh in HIV positive women decision of motherhood. In this context, it is necessary to provide these women with the necessary counselling, guidance and resources to take decisions about procreation properly informed.

**Electronic supplementary material:**

The online version of this article (10.1186/s12905-017-0483-y) contains supplementary material, which is available to authorized users.

## Background

The HIV/AIDS epidemic in Spain is concentrated in men: of the 3353 new HIV cases reported to the Spanish New HIV Diagnoses Information System (SINIVIH) in 2016, only 16% were female [1]. This differential distribution is also found in the data provided by the European Centre for Disease Prevention and Control (ECDC) that showed a male-to-female ratio of 3.2 in the European Union/Economic Area (EU/EEA) in 2016. However, absolute numbers reveal an important epidemic of HIV among females: from 2007 to 2016 there have been 170,530 women diagnosed with HIV in EU/EEA [2].

In recent years vertical transmission of HIV -from mother to child- (MTCT) has dropped sharply. This is mainly due to the improvement in combination antiretroviral therapy (cART), scheduled caesarean deliveries and the use of assisted reproductive technology (ART). In Spain only 0,2% of new HIV diagnosis were acquired by this route in 2016 [1].

Therefore, women living with HIV are increasingly considering having children. Pregnancy is perceived by women living with HIV as a “way to regain their sense of womanhood and sexuality” after HIV diagnosis [3]. Recently, a cross-sectional study with HIV positive women of childbearing age from the Spanish AIDS Research Network Cohort (CoRIS) revealed that desire for children in these women was frequent (49%) [4] and pregnancy after HIV diagnosis was also common (39%) [5]. This study also showed that main reason for procreation desire were that women cherished children or wanted to start a family [4]. Expressed barriers to motherhood among these women were HIV infection, age or having already had children. Additionally, other research has documented several barriers related to HIV infection: fear of vertical transmission or of leaving an orphaned child due to the mother’s infection [6]; HIV stigma and social rejection of people living with HIV [7, 8]. Gaps in information provided by clinicians to women living with HIV on guidelines and methods to have safe pregnancies have also been previously identified [9]. Described barriers make difficult to reach an intimate partner relationship and to enjoy a full sexual life among these women.

However, research conducted in developed countries suggest that factors related with the decision to procreate in HIV positive women are similar to those among general population and mostly determined by socio-cultural and personal factors than by other aspects strictly related to HIV [7, 10–15].

The World Health Organization (WHO) has underlined the need to address sexual and reproductive health of women living with HIV/AIDS in order to ensure both their welfare and that of their partners and children [16].

Little research on this topic has been carried out in our context, apart from the study performed by Hernando et al. previously mentioned. Furthermore, that study was quantitative and missed an in-depth analysis of sexual life and factors related to desire for procreation in HIV positive women. These circumstances led us to design a qualitative research whose results are presented in this article. To our knowledge, this is the first study in our setting aimed at acquire a thorough knowledge of sexual life and elements that shape desire for procreation of women living with HIV and analyse HIV diagnosis impact on both. This first article presents results related to motherhood and desire for procreation.

## Methods

### Theoretical framework

**Fig. 1:**
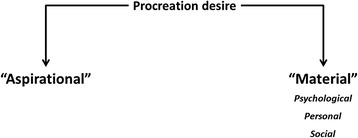
Conceptual framework

Conceptual scheme of our study is presented in Fig. 1. Accordingly, desire for procreation of women consists of material and “aspirational” elements (motherhood “social construct”) including social, psychological and personal factors. This framework contained the following elements:

- “Aspirational” elements related to motherhood. Maternity’s social projection (maternity as a social identity value for women). Meaning of “Motherhood” as a “concept” (objective) and as “experience” (subjective).
- Material elements that make up the desire for procreation. Barriers and drivers of procreation:
  The partner: the importance of a partner in motherhood decision, having a partner as well as a co-decision maker.
  Factors influencing motherhood decisions: with who is shared the motherhood decision.
  Other items related to desire for procreation.

### Method and instrument of data collection

A qualitative study was conducted through open-ended interviews to women between 18 and 45 years of age, included in the Spanish AIDS Research Network Cohort (CoRIS).

CoRIS is a cohort of people aged 13 years and above, HIV positive, who have never taken antiretroviral treatment and are new for following up in participating centres (28 hospitals and medical centres across Spain [17]).

To develop the interview an open script based on the literature review done previously was preferred. The script is showed in Additional file 1 and contained the following areas:

- Impact of the diagnosis on the sexual, emotional and reproductive life of the women. Changes in women’s talks about their self-perception after and before HIV diagnosis.
- “Aspirational” elements related to motherhood.
- Material elements that make up desire for procreation.
- Script was piloted and little modified with the first interviews.

### Sample design

In 2010 we conducted a quantitative survey about reproductive history and desire for procreation in 134 women participating in CoRIS of nine hospitals and one sexual health clinic [4]. For the qualitative study, a subsample of 28 women was randomly selected among those surveyed in the quantitative phase. Twenty of them agreed to participate in the qualitative survey. Among non participant women main reasons were fear of HIV disclosure in their social circle and being away at the time of the study.

Sample was segmented according to the following variables: desire for procreation (yes/no), have previous children (yes/no) and country of birth (Spain/other). To allow for sample heterogeneity, we also took into account other variables such as having a partner (yes/no), women’s age (under 35 years old/35 or more) and educational level (primary or none/secondary or higher).

### Participants’ recruitment and consent

Clinicians involved in the study selected women and introduced to them the project. We then approached women by telephone and scheduled an appointment for the interview. Before interviews, participants signed a specific informed consent for this qualitative study. Participants only knew about researchers that team was involved in the Spanish AIDS Research Network Cohort. They participated in the first phase of the research (quantitative stage), then they possibly would anticipate the interviews subject. No information about interviewers’ characteristics was anticipated to them.

### Interviews development

Interviews were conducted between May and July 2013 by a researcher skilled in qualitative research studies in our team (Dr. Alvarez-del Arco), occasionally accompanied by another researcher (Dr. Hernando). Nobody else was present during the interviews. Interviews were performed at the place selected by the patient (her home, the hospital or other medical settings or elsewhere). Interviews were audio-recorded and had an average length of 80 min. None of interviews was repeated. Data saturation was discussed among researchers although resources limitations did not allow us for increasing the number of interviews.

### Method of analysis

After interviews field notes were made and a literal transcription of audio recording content was developed. Transcriptions were not returned to participants for comment and/or correction, since it was logistically complex.

We analyzed data using a content analysis approach. Following this approach, some of our codes derived from our theoretical framework and other were directly derived from the retrieved data. To do this, two researchers independently encoded the data using the “Open Code” software [18] that allowed us to better organize data within transcriptions. Codes were discussed between both researchers and agreements were reached among them. We interpreted codes taking into account the underlying context and participants’ characteristics.

Analytical dimensions that grouped codes were generated from the code list and reviewed and discussed with the rest of the team with experience in qualitative analysis.

### Ethical approval

The study was approved by the Instituto de Salud Carlos III Research Ethics and Animal Welfare Committee of the (Madrid, Spain).

### Research team

#### Experience and training

Debora Alvarez-del Arco is Sociologist and PhD in Medical and Social Sciences (University of Alcala), Specialist in applied Social Research (Centro de Investigaciones Sociológicas) and Expert in Migration, Exclusion and Social Integration Policies (Universidad Nacional de Educación a Distancia). She has 14 years of working experience in social and market research companies, developing qualitative researches.

#### Gender composition of the team

Female researchers in our team were Alvarez-del Arco D, Rodríguez S, Pérez-Elías MJ, Cuellar S, Masiá M, Pascual L, Hernando V. Male researchers were Blanco JR, del Romero J, Santos I, Boix V.

## Results

Of the 20 participating women, nine were Spanish and 11 foreign, six of them from Latin America. All contracted HIV through heterosexual contact, through their steady or casual partners, except a woman who was infected through occupational exposure. Mean of years since HIV diagnosis among these women was 7.8 years. Women’s socioeconomic and clinical characteristics interviewed are summarized in Table 1.

**Table 1:** Characteristics of participant women

No.	Age	Country of birth	Study level	Have partner	Have Children	Procreation desire	Year HIV diagnosis	Employment status	Post HIV pregnancies
1	39	Spain	Secondary or higher	Yes	No	No	2008	Employed	No
2	34	East Europe	Primary or none	No	No	Yes	2004	Employed	No
3	37	Spain	Primary or none	Yes	Yes	No	2002	Unemployed	Yes (abortion)
4	47	Spain	Primary or none	Yes	Yes	Yes	2003	At home	No
5	34	Latin America	Primary or none	Yes	Yes	No	2004	Employed	No
6	28	East Europe	Secondary or higher	Yes	No	No	2009	Employed	No
7	41	Spain	Primary or none	Yes	No	Yes	2007	Employed	No
8	29	East Europe	Primary or none	Yes	Yes	Yes	2007	Employed	Yes (diagnosis during pregnancy)
9	47	Latin America	Primary or none	Yes	No	No	1987	Employed	No
10	42	Spain	Secondary or higher	Yes	Yes	Yes	2008	Unemployed	Yes (Ass.reprod.tech)
11	34	Spain	Primary or none	Yes	No	Yes	2008	Unemployed	No
12	43	Spain	Primary or none	Yes	Yes	No	2007	Unemployed	No
13	45	Latin America	Secondary or higher	Yes	Yes	No	2009	Student	No
14	36	East Europe	Primary or none	No	Yes	No	2005	Employed	No
15	45	Latin America	Primary or none	Yes	Yes	Yes	2008	Employed	No
16	34	Sub-Saharan African	Primary or none	Yes	Yes	Yes	2005	Employed	Yes (diagnosis during pregnancy)
17	37	Latin America	Primary or none	No	No	Yes	2001	Employed	No
18	34	Spain	Primary or none	No	No	No	2004	Student	No
19	22	Latin America	Primary or none	Yes	No	Yes	2009	Unemployed	No
20	42	Spain	Basic	Yes	Yes	Yes	2004	Retired	Yes (abortion)

We summarize our findings organized in the following topics: a) The impact of HIV diagnosis; b) The concept of “motherhood”; c) Dimensions of motherhood. Some of themes presented in this section were advanced in our theoretical framework and other derived from the retrieved data.

### The impact of HIV diagnosis

Stigma associated with HIV appeared when women narrated when they found out their HIV status for first. They expressed a number of feelings: fear, guilt, self-rejection, isolation. In summary, HIV diagnosis was experienced as a turning point among participant’s lives.

Firstly, HIV diagnosis changed women’s self-perception and some of their point of view about life key topics. HIV disclosure was experienced as a traumatising experience and women associated HIV diagnosis with the idea of an immediate death.

*"... It’s an awful situation. When you didn’t know it and you are told you are HIV positive you think, “I am dying”... you say “I am going to die”. But then...you realise that you will be able to move forward. But you go through a terrible, very terrible time. I mean, it’s a psychological shock... horrible, horrible... and when you think why it has happened (...), because of a mistake, by going bed with someone and not to take protection ...”.* Interviewee No. 7.

Altogether, among participant women there was a sense of guilt associated with HIV acquisition. They become HIV positive through unprotected sexual intercourses and they considered it a mistake they were responsible for. These guilty feelings and regrets were expressed by women in different ways. For example, one of them described a distorted image of herself (“a virus shadow around her body”) linked to self-rejection derived from HIV diagnosis.

*"For about three or four months [after HIV diagnosis] when I looked at myself in the mirror I could see around me the shadow of the virus (...). It was horrible, because I was disgusted by myself. I looked at myself in the mirror and I could see around me, literally, a grey shadow ... And I thought “this is the virus”".* Interviewee No. 11.

Lack of self-acceptance right after HIV diagnosis was expressed through speeches about three main aspects: sexual life, couple relationship and motherhood. Regarding sexual life, women perceived themselves “non-sexual subjects” due to fear of HIV transmission to their partners and such as a rejection of the way in which most of them acquired HIV. Regarding couple relationship, women considered their partners would abandon them or, if they lacked a partner at the moment of HIV diagnosis, they would never find one. Last, regarding motherhood, women believed they would not be able to deliver HIV-free children.

This women’s self-rejection gradually disappeared at the same time that HIV status was accepted. However, a clear turning point remained in women speeches: the pre-HIV life and post-HIV life. In addition, some of the elements related with HIV stigma Remained, for example, social isolation after diagnosis. Strong stigma associated with HIV forced women to systematically hide their serostatus. HIV status was disclosed only to a closed social circle and fear of HIV disclosure was frequent.

### “Motherhood” concept

**Fig. 2:**
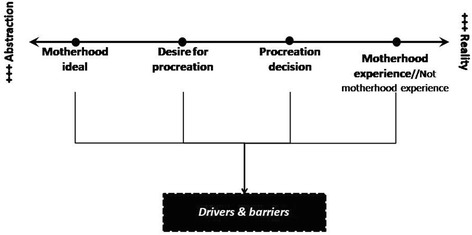
Dimensions of motherhood

Interviews shown that “Motherhood” was a complex concept for women. Actually, the concept of “motherhood” is shaped by “objective” (external, exogenous) and “subjective” (internal, endogenous) dimensions. These aspects derived from both, women’s subjective experiences as mothers (real experiences) and from social representations of motherhood: social values, beliefs, ideas, beliefs and practices related to motherhood shared among members of the same social community of participant women (social expectations). These findings increased the complexity of the “theoretical framework” initially designed: in this section, motherhood representations and experiences will be analysed in four different dimensions according to their level of abstraction (Fig. 2).

The first dimension was the “Motherhood ideal” (social representation / archetype of motherhood). This dimension could be defined as the "social imaginary that shaped women’s conception of maternity", since it was configured by intangible elements and closely linked to the “aspirational ideal” of motherhood. This social representation is shared by women belonging to the same society.

The second dimension was the “Desire for procreation” built by different social, psychological and material factors, closely linked to the “material” level of our previous “theoretical framework” designed before the research.

The third dimension was the “Decision on motherhood,” the moment when women decided to make desire for procreation come real.

The fourth and last dimension was the “Experience (or the lack of experience) of motherhood”, and it was represented by the current experience of maternity (or the lack of it).

### Dimension 1: “Motherhood ideal”

The idea of an “Aspirational” dimension of maternity including social representations was represented in the original framework. However, interviews showed it was a culture-specific dimension and allowed for a better understanding on how women built this ideal. “Motherhood ideal” was closely linked with ideals of femininity and with the feel of “being a complete woman” throughout having children. In addition, motherhood was conceived as a life process that transcended mothers’ lives: progeny was perceived by women as a self-prolongation after death.

Intangible and culture-specific elements defined archetype of maternity internalized by women. “Ideal of motherhood” was associated with “being a woman”, was legitimised as an “instinct” and was perceived as something that “completed” women:

*"... Having a child is ... is ... is ... something indescribable. It fills you with all you need... But, [having children] is an achievement".* Interviewee No. 13.

Women who rejected motherhood as an element intrinsically linked to femininity explicitly or implicitly recognised this pattern of thinking as socially predominant. For women, motherhood ideal was closely linked to the idea of a traditional family settled in life-course early stages: a family consisting of a couple and several children. Actually, motherhood was considered as the most important demonstration of love for the partner.

*"*[I wanted to have a child ...] *because I was crazy in love with my husband, completely crazy ... and I wanted to have a child with him. Because I knew he would never leave me, because we're going to be together forever. It’s going to be... next October, twenty years that we're together.”* Interviewee No. 4.

As previously stated, this dimension is linked to a specific social notion about what is motherhood and is not related with women’s personal situation. Because of this, this “Motherhood ideal” did not change after HIV diagnosis. However, this ideal has consequences at the time of diagnosis: since women thought they could no longer have children, they considered them as “incomplete women”. As before pointed out, this perception was transitory and generally changed with increased knowledge about HIV.

*“*[She was reproducing a talk with her husband after HIV diagnosis] *I will be useful for you nevermore... I will not be longer a woman, no, I won’t be of any use to you, I will be not able to give you children ..."* Interviewee No. 5.

*"The first question was if I would be able to have children, it was the fear of first weeks, when you have little information about what is happening to you (...) That was my main fear: to be able to have children, you know, until I improved my knowledge about HIV. So when you learn that it is feasible and you can move forward, that is an injection of joy, from thinking it was impossible to knowing that it will be possible”.* Interviewee No. 10.

### Dimension 2: “Desire for procreation”

Women shaped their desire for procreation through a balance between reasons and barriers for reproduction.

### Reasons for becoming a mother

Women found it complicated to verbalize reasons for wanting to become mothers. Arguments supporting procreation desire among participants were diffused and usually based on imprecise elements: women’s “maternal instinct”, the fact they “like children” or in a transcendental perception of “destiny”, something that they, as women, had to do.

When they managed to verbalize reasons for procreation, partners’ desire were key. When the partnership was enough consolidated, women considered having children was a love demonstration. When this love demonstration was aimed to someone living with HIV, some women believed it was even more significant:

*"*[She was talking about the importance of her husband on the decision on motherhood] *... At that moment I felt he really loved me. I felt he really loved me because despite of consequences [of my HIV infection] he wanted a child with me. He didn’t doubt ... “And if it goes wrong?” or “With this woman not be worth it”. “But he had not thought it, he wanted to go forward. I really think it means a lot for me. ".* Interviewee No.5.

Women also expressed other elements related with the desire to become mothers: a double need: having children meant having someone to care for and also to have someone that would be a caregiver for them in the future. Women wanted to have someone to love, to cherish and someone to fight for:

*"Having a baby is (...) to have someone to take care of, having someone to give lot, and lot of love, have someone to educate (...) then … joy he can give you..."* Interviewee No.11.

In addition, they also expressed need to guarantee their own care and support in the later life stage, considering children as an investment for future.

"*But you have to have children! (...) When you're old, who will take care of you, who will love you, who...? Well, I need to have a baby ".* Interviewee No. 11.

### Barriers to procreation

Among women living with HIV there are generic barriers to procreation and other specifically related with HIV infection. In this study, women verbalized the following generic barriers: lack of a partner, sacrifices derived from becoming a mother and economic stability lack. Among the barriers related to HIV we found: fear of having children infected with HIV, concerns about self-health and ability to childcare and social perceptions of motherhood of women living with HIV.

Lack of a partner in the procreation process meant lack of emotional, logistical and financial support. The option of being single mother was right perceived, but only few participants considered it as a realistic option.

*"... It is important having the two figures for the baby, both maternal and paternal. If I was single I certainly would not consider it. Mostly because you need time for the baby, I do not want to leave the baby either to my parents or in a nursery school. You cannot work and have a child at the same time. I would certainly not consider it if I was single".* Interviewee No.11.

Motherhood was considered an experience resulting from a life choice in which mother “sacrifices” her personal life for caring children. Some participant women argued they did not have enough “maternal instinct” to compensate this “loss”.

On the other hand, the lack of economic stability was one of the most frequently mentioned barriers to procreation. However, it seemed to be a real barrier only among those with financial constraints. Some women used this argument to justify not wanting children and to face with a social perception that linked women with motherhood.

There were other generic barriers related with previous experiences or health problems. Having already lived the experience of motherhood or women’s age were critical to relinquish new motherhood. In addition, negative experiences in previous pregnancies and having other health problems unrelated to HIV were also included among barriers to procreation.

HIV was not explicitly expressed as an obstacle to motherhood: women were healthy and perceived medical improvements would protect children from HIV. HIV appeared unconsciously in women talks: a latent fear of transmitting HIV to the baby and, also, in the fear of becoming ill and consequently, unable for child caring.

*“(...) It is very hard to explain, but it’s something very intimate (...), you’re pregnant, you know you won’t transmit the infection, because you know that nowadays retroviral treatment protect them from HIV if you take your medication... But it doesn’t matter, if you have accepted this responsibility with the infection you have, you will have it [the fear of infecting the baby]. (...) And you say, “is there a need for this?” I would be very selfish, you say: “What need do I have to bring a little person into this world with my circumstances if maybe tomorrow I’ll need special care... is it necessary..?. Is my need to be a mother so great that I will put a person through this?”*. Interviewee No.18.

To face this HIV related barrier to procreation, some foreign women showed their religious believes by considering God would protect them and their babies from HIV. This role of religion has not been found among Spanish women.

*"... And if I take care of myself I think maybe God will give me a lot more time of life. Because I pray to God everyday: “God, give me life, I want see my daughters to grow up with me, I want them to see me well, I want myself being able to work ".* Interviewed no. 5.

Among the few women who were against motherhood with HIV, arguments were focused on the fear of delivering children infected with HIV. Once again, women’s guilt sense appeared in their talks: some of them mentioned their own responsibility to protect children from their mothers’ “mistakes”.

[She was talking about the possibility of having children after HIV diagnosis] *"With my way of thinking ... No, God, no. It is not because of the other person, for example, of my partner (...), but the child (...) Imagine that the child has to take treatment (...)just in case... (...) why should the child have to suffer because of my silliness? (...) no, no ... No way ...”* Interviewee No.14.

HIV strong stigmatization also appeared among reasons to give up motherhood. Social perception about motherhood of HIV positive women was key: they considered society believed children of mothers with HIV born HIV infected. In this context, there was a social perception of motherhood as a selfish decision of women living with HIV.

### Dimension 3: “The decision of motherhood”

Motherhood could be both, a complex decision or an unplanned event. In the cases of unwanted pregnancies, decision on motherhood was taken after pregnancy. In these situations, pregnancy is the result of an inconsistent use of contraceptive methods which reasons have not been specifically explored. In these cases, some women went ahead with pregnancy while other explained how they underwent an abortion:

*"...* [She was talking about how she became pregnant shortly after meeting her partner] *At first we were supporting the idea of having the baby, but then I backed down. I was scared, I had known [my partner] for a month, I knew what to raise a child alone was..., the cost, not only the money but also the education (...) I could not to take the risk. A child is for all the life... ".* Interviewee No. 20.

When motherhood was a planned event, women intentionally stopped using contraception or underwent fertility treatments to have children. In this decision of “intentional” motherhood two elements have an important influence: partner’s opinion, which has been already analysed, and clinicians’ advices. HIV made decision about motherhood more difficult: women thought about it meant an added difficulty to the experience of became a mother. Therefore, clinicians’ counselling about how to deliver safe children and how to protect partners from HIV was essential in their decisions.

"*But above all, we have, we have asked [the doctors] about it (...). We asked if it would happen [a pregnancy] what will be the risks (...) you know? For both the unborn baby and me, that is, what would happen, because, of course, it scares you ... ".* Interviewee No.7.

*"Without the doctor there’s nothing, because the doctor is the one who decides: “Well let’s go for it. We should do it in this way.” If the doctor tells me, “Jump off and everything will be right” I will jump. I will only do what the doctor says".* Interviewee No.5.

Nevertheless, not all clinicians systematically provided with information about how to minimise risks in the proration process and during pregnancy. Many women had to proactively ask clinicians for this information. Other participants preferred to look for information since some of them experienced barriers to directly ask doctors about this topic.

"*No* [the doctor hasn’t given me any information], *I come to appointments, and he says “You are o (...). Are you taking your medication? (...)”*. *What I know* [on how to safely get pregnant] *I found it by myself on the Internet or reading or things like that".* Interviewee No.17.

The need of information was different depending on women’s and their partners’ circumstances. For example, in the case of serodiscordant couples, provide them with knowledge about assisted reproductive technology (ART) was essential. However, ART was perceived as an unnatural procedure.

*"Well, the first thing that we discussed* [with the doctor] *was related with assisted reproductive technology (...)when I heard for the first time about this disease the first thing I asked him was whether I would be able to have children (...) and at all times he spoke about assisted reproductive technology , so, I have always thought that I would have a “tinned” baby... as I call them, you know? “.* Interviewee No.7.

Actually, couples who had no other health or fertility problems than HIV preferred to have natural pregnancies, using the only protection of antiretroviral therapy.

*"... since I already get the medication, they* [clinicians] *refereed me to start taking it, they said: “you have to have normal sexual intercourses and get pregnant”. Then, of course, that is an excitement....".* Interviewee No. 10.

Use of ART was finally restricted to few couples, which decided to use is because of health problems unrelated to HIV. In these cases, to get pregnant was a long and very complex process. In addition, women undertaken it considered that being HIV positive affected the response received from health services in charge with this technology, adding for one of participant women, a new barrier to procreation.

*"... we came here at some point* [to the hospital]*, what happened when we came here... I don’t know ... they had no previous experience treating HIV-positive women, and they didn’t give us any information. And it was awful... now I think it has changed. That was four or five years ago. You had no information, it was my first time...and we were asked weird questions, as if we were weird ... ".* Interviewee No. 1.

Women undergoing assisted reproductive technology described it as a long process. One of participants described frustration derived from unsuccessful ART treatment.

*"I have been on* [ART] *treatment for something like five years... all types, all injections you can imagine and everything, everything, everything, everything, everything ... I do not know how many treatments I underwent (...) And my husband said: ‘This is as far as we are going, because you are losing yourself’ it was very frustrating to be inseminated and then nothing... and then undergo to another one (...). I decided to not do it* [the sixth insemination]*, I said: “this is as far as I am going, (...), no ... I cannot do it anymore".* Interviewee No. 4.

### Dimension 4: Experience (or the lack of experience) of motherhood”

Real experience of maternity had an important impact on women lives since it was considered as a step into the adulthood stage of life. In addition, participants experienced motherhood as a chance to come back to their pre-HIV life and as an element to compensate other traumatic life-events they experienced in the past. Among those participants who did not want to become mothers there was the need of feeling that there was still time to take the final decision about motherhood.

Women experienced motherhood considered it as a step into adulthood. They thought it was a stage of life in which new challenges and responsibilities would be taken. Participants told it was a moment that brought out their insecurities concerning their own worth as mothers. In fact, women with previous children enjoyed more the following motherhood than the first.

*"... It's not like when you are twenty five years old. I did not enjoy my* [first] *daughter (...) because then I worked more than a fool. My mother brought her up... my mother and my sister. I was always working. So now I think I would enjoy it more... ".* Interviewee No.20.

Motherhood was an element that made those women feel themselves “normal despite HIV” and helped them to overcome HIV diagnosis trauma. In addition, there were some women suffered in the past high vulnerability situations (prostitution, alcohol or drugs abuse, harmful partner relationships, rapes…) that considered motherhood as a personal compensation. In fact, after having children, many of them changed their lives to avoid these situations. These women said they regained their self-esteem through their own recognition as women and as mothers. Thinking that they could be “good mothers” generated self-esteem, and they assumed their own care responsibly (taking antiretroviral treatment, visiting a clinician) not for their own benefit, but to be able to take care of their children.

*"If I did it* [take the cART] *it was ... it was for my girl, I wouldn’t have taken them for myself..."* Interviewee No.19.

HIV caused women’s self-consideration as “special mothers”. Firstly, social stigma associated with HIV gave them an additional responsibility: to ensure that their HIV status remained hidden to protect children from this stigma. On the other hand, they considered themselves as “incomplete mothers” because they could not breastfeed their children. In this context, partner and family support, when aware of woman’s serostatus, was key in her motherhood experience.

Among women who had not had children it resulted from a women’s voluntary decision, from women’s health problems or derived from life circumstances that prevented motherhood. Women who had voluntarily decided to not become mothers reaffirmed their decision, but needed to feel they were still able to become mothers in the future.

Women who had not the chance to became mothers, this lack was experienced with frustration. Some of them felt they had experienced some aspects of motherhood through other people’s children and even through the care of pets.

*"I know I can be a good mother ... (...)I have not been a mother because I have not given birth but... my brothers, I’ve brought up them... food, clothing ... help my mother, I have been the one ... the one that has been there ... ".* Interviewee No.17.

## Discussion

This paper analyses procreation desire of women living with HIV in four different dimensions: the ideal of motherhood, the desire for procreation, the decision of motherhood and the experience of maternity. We specifically study the role of HIV in each of these dimensions. Findings of our research allow us to improve knowledge about the complexity of this topic and show how women living in Spain with HIV suffer latent, but important, barriers related to HIV salient in the final decision about maternity.

This qualitative study adds knowledge to the Hernando et al. [4] previous study, which showed desire for procreation in HIV-positive women in Spain was prevalent, as was also demonstrated by research performed in other European countries [3, 19]. Our study allows for an in depth understanding of barriers and drivers for procreation in HIV positive women, and impact of HIV infection on them.

In this article we show how most of drivers for procreation among participants have nothing to do with HIV infection. However, participants acknowledge that learning that it is possible to prevent mother to child-transmission encourage them to have children. Two main reasons for procreation were revealed by the previous quantitative study and also found in Spanish general population [20]: love for children and desire to start a family. Our qualitative research shows importance of partner in the decision of having children, as was also observed in the quantitative phase [4] and in other studies conducted in developed countries [21, 22]. Participants of our qualitative study perceive children as the most important love manifestation to the partner. In addition, partner is considered a source of logistical and financial support. Need to have someone to love and care for and need for have someone to provide women with care and company in their later stages of life complete reasons for procreation among our participant women.

In our study HIV indirectly appear among participant’s barriers to procreation. Main expressed impediments to motherhood are lacking of economic stability and lacking a partner, in line with those found in general population in Spain [23] or in women living with HIV in the United Kingdom [24]. HIV-related barriers are latent in women’s speeches. In this regard social stigma associated with HIV plays a key role. Participants perceive public opinion is against motherhood of HIV positive women. Social rejection is a significant barrier for procreation among them and it has been already identified in the United States’ (US) researches [25, 26]. HIV social stigma makes women to systematically hide their HIV status in order to protect their children and themselves, from social rejection.

Apart from stigma associated with HIV, women face a fundamental barrier to procreation associated with their serostatus. Participants suffer before and during all the pregnancy fear of infecting the baby. Haddad et al. showed how in US HIV positive women were concerned for horizontal and vertical HIV transmission when deciding if having children [27]. Recent research have shown how improvements in the management and treatment of HIV [24] and in the women’s awareness about vertical transmission risks contribute to an increased desire for procreation [21] and to increase the number of women deciding to have children after HIV diagnosis [13–15]. In this sense, our study has shown the importance of health workers throughout the whole process of motherhood in women with HIV, both, as support and as information providers. However, our study shows similar findings to those presented by Finoccahrio-Kessler et al.: there are communication barriers between “provider” and women when addressing reproduction issues and sometimes women is who have to initiate the discussion on these topics [28].

In relation to assisted reproduction technology, in our study these treatments are considered as an option only for those few women with other concomitant health problems. In Spain, women with HIV have free access to this type of techniques [29, 30], although they may face other structural barriers to such access. In fact, our study shows also how women with HIV are afraid about HIV implications for their rights to use these services or in the personal treatment received from the medical staff. In concordance with the study of Loutfy et al. performed in Canada [9], our study reflects the importance of well-informed clinicians discussing with patients and partners their pregnancy plans.

Our study shows HIV diagnosis has a strong, but transient, initial impact on women’s lives. This is a traumatic life-event manifested in a temporary distortion of women self-perception and in the motherhood self-denial. After this phase, desire for procreation among women with HIV remains and ideal of motherhood and social values ​​associated with it remain unchanged. These women perceive motherhood as an element that will normalize their lives and will help them to cope and psychologically overcome the diagnosis. Motherhood is experienced as a process of empowerment where women take the responsibility of their own care to ensure the care of their children. In this sense, other research has shown motherhood is a highly valued social identity [25] that helps women to regain their self-esteem [26]. In fact, women in our study consider motherhood as a compensating element after HIV diagnosis.

Finally, our research presents a number of methodological limitations that we have to acknowledge. Firstly, all participating women are followed in hospital or clinical centres. Therefore, HIV positive women in high vulnerability situations that are not accessing health services are not represented in our study. Time and budget constraints have not allow us for developing a study with consecutive in-depth interviews with every woman. This methodological approach would have been more appropriate in order to address an issue so sensitive and intimate such as the presented in this article. Similarly, we have not investigated the perceptions of men with HIV or partners of women with HIV about this topic. Include their testimonies would help us to complete knowledge on HIV impact on subjects’ reproductive decisions, allowing to assess and to understand gender axis in desire for procreation.

In addition, there is not much scientific literature about this topic available on our context. Most of cited research to provide scientific evidence to support our results are studies conducted in Western countries whose study’s populations differ from ours. These studies have been mostly done in women from marginalized social groups -Afro-American, Hispanic, aboriginal or migrant women with lack of economic resources- [6, 10, 12, 14, 24, 25]. Although many of the participating women in our study had experienced situations of great vulnerability in the past, nowadays they are fully social-integrated. The unique distinguishing feature of some of the women in our sample were references to God role in their HIV experience. It has been also observed in US’s studies among African-American women [11, 31].

## Conclusion

In conclusion, stigma and discrimination towards people with HIV is perceived as stronger against HIV positive women who decide to have children. Social stigma weighs in women living with HIV’s motherhood choice. For them, family and partner are an important support. In this context, health professionals also play an important role in those women reproductive decisions.

Our findings have important implications for healthcare services that both health providers and policy makers should take into account. This study shows a gap in the women living in Spain with HIV needs. Firstly, HIV had an important impact on women’s desire for procreation: they feared HIV consequences in their children, not only at health level, but also related with social stigma they have to deal with. Secondly, this study highlights clinicians should proactively approach women of reproductive age to inform them how to safely have children. In addition, psychological support has to been provided together with targeted reproductive assisted services.

It is therefore necessary to provide women with HIV the necessary counselling, guidance and resources to make decisions properly informed about their sexual life and procreation.

## Additional file

Additional file 1: “Interview script”. Interview script used to develop the interviews. In the Additional file 1 we included both, the original Spanish version and the English translation of the script. (DOCX 20 kb)

## Abbreviations

ART: Assisted reproductive technology
cART: Combination antiretroviral therapy
CoRIS: Spanish AIDS Research Network Cohort
ECDC: European Centre for Disease Prevention and Control
EU/EEA: European Union/Economic Area
MTCT: Mother to child transmission
SINIVIH: New HIV Diagnoses Information System
WHO: World Health Organization
