# Supplementary material for: Role of HIV in the desire of procreation and motherhood in women living with HIV in Spain: a qualitative approach
Source: BMC Womens Health. 2018 Jan 24;18:24. doi: 10.1186/s12905-017-0483-y (PMC5784503; doi:10.1186/s12905-017-0483-y)
Supplement: Additional file 1: — “Interview script”. Interview script used to develop the interviews. In the Additional file 1 we included both, the original Spanish version and the English translation of the script. (DOCX 20 kb) [file 12905_2017_483_MOESM1_ESM.docx]

**Additional file 1. Interview script**

**English translation**

I. Background: reproductive history

In the interview we did by phone you told us that you had children / you did not have children ... please, tell me a little more about this ...

II. Social projection: the meaning of being a mother (objective/subjective perspectives about motherhood).

- Maternity as a “social value of identity” for women.
- The meaning of motherhood as "concept" (objective).
- The meaning of motherhood as "experience" (subjective).
- Self-esteem.

III. Elements shapening desire for procreation

As you may know, women often decide whether to have children weighing the pros and cons ... what would you put in your balance of pros and cons?

IV. Partner’s role

The importance of having a partner in motherhood experience: conceptually and as a co-decision-maker.

When thinking about the ideal partner everyone considers different elements to take into account. I would like you to tell me what things are important to you in a relationship ... (introductory). And…how important do you think sex isin a relationship?

- Meaning of sexual life (how they live and conceptualize sexual relations ...)

V. Social agents influencing on motherhood decision and HIV disclosure.

- Sometimes, when we have to take a decision we share it with someone, to whom we ask for their opinion. With whom did you share your decision to be a mother / not a mother?
- Who has weight in the decision making: society-doctor-family-partner
- Who knows you are HIV positive?

VI. Change of speech before and after HIV diagnosis: her reproductive history

- How did HIV affect you in your decision to want children / notwant children? What do you believe people think about a woman with HIV wanting to have children? (projection).
- Other changes after HIV diagnosis: relationship, sexual intercourse, contraceptive methods…. What changed after your HIV diagnosis? How didyou change it?

VII. Other elements related to the desire for procreation:

- Knowledge and attitudes towards HIV.
- Spiritual beliefs.
- Psychological and reparative elements: generating self-esteem, self-esteem, failure to raise other children, need for having someone to give unconditional love, need for having someone to exercise control.
- Personal stigma about HIV: having a child means to reproduce stigma?

**Spanish original version**

. Antecedentes: historia reproductiva

En la entrevista que te hicimos por teléfono nos dijiste que tenías hijos / no tenías hijos. por favor, cuéntame un poco más sobre esto ...

II. Proyección social: el significado de ser madre (perspectivas objetivas / subjetivas sobre la maternidad).

- La maternidad como "valor social de identidad" para las mujeres.
- El significado de la maternidad como "concepto" (objetivo).
- El significado de la maternidad como "experiencia" (subjetiva).
- Autoestima.

III. Los elementos que conforman el deseo de procreación

Como sabes, las mujeres muchas veces decidimos si tener hijos poniendo en una balanza los pros y los contras… ¿qué podrías tú en la balanza de los pros/qué pondrías en la balanza de los contras?

IV. La pareja

La importancia de la pareja en la maternidad: en abstracto (tener pareja) y también como co-decisor.

Ya sabes que a la hora de pensar en nuestra relación de pareja ideal existen una serie de elementos a los que concedemos mayor o menor importancia en función de cómo sea nuestra forma de pensar. Me gustaría que me dijeras qué cosas son importantes para ti en una relación de pareja… (introductorio). Y ¿el sexo? ¿Cómo de importante crees que es?

- Significado de la vida sexual (cómo viven y conceptualizan las relaciones sexuales ...)

V. Los agentes que influyen en la toma de la decisión sobre la maternidad

- A veces, cuando tomamos tenemos que tomar decisiones las compartimos con algunas personas, a las que pedimos su opinión. ¿con quién/es compartiste tu decisión de ser madre/no ser madre?
- Quién/es pesan en la toma de decisión: sociedad-médico-familia-pareja.
- Quién sabe que es VIH positiva.

VI. Cambio de discurso pre-post VIH: a través de su historia reproductiva

- ¿Cómo te afectó el VIH en tu decisión de querer tener hijos/no tener hijos?¿qué crees que piensa la gente sobre que una mujer con VIH quiera tener hijos? (proyección).
- Otros cambios después del diagnóstico del VIH: relación de pareja, relaciones sexuales, métodos anticonceptivos🡪¿Qué cambia? ¿cómo lo cambia?

VII. Otros elementos relacionados con el deseo de procreación:

- Conocimientos y actitudes frente al VIH.
- Creencias espirituales.
- Elementos psicológicos y de reparación: generación de autoestima autoestima, fallos en la crianza de otros hijos, necesidad de amor incondicional, alguien sobre el que ejercer el control.
- El estigma personal sobre el VIH: (tener un hijo significar reproducción del estigma.
